# Society Meetings

**Published:** 1915-05

**Authors:** 


### SOCIETY MEETINGS

Brief reports and announcements of meetings of societies of Western New York, and those of general scope, are requested from Secretaries. Copy should be on hand the fifteenth of the month. Full transactions will be published at cost of composition.

**DOCTOR: Is your Society properly represented here? If not, please bring our standing announcement to the attention of the members.**

**Medical Society of the State of New York.** In the last three issues, we have devoted considerable space to announcements of the meeting at Buffalo. Now that the meeting has passed into history, we feel that it is both expedient and courteous to leave the reports to the official organ of the Society. Every member of the profession ought to be affiliated with the State and County organizations and, therefore, de facto, a subscriber to the State Journal. Our failure to duplicate matter appropriate to this journal is therefore, not to be construed as a lack of interest but rather a desire to serve the interests of the organized profession.

**The American Gastro-Enterologic Association** will hold its eighteenth annual meeting May 10 and 11, in Osler Hall, Johns Hopkins University, Baltimore.

**The Elmira Academy of Medicine,** March meeting. J. A. West, D. D. S., read a paper on Oral Sepsis. Dr. Bell reported a case of poisoning from the injection of ¼ grain of morphine. The patient stopped breathing within 10 minutes but was out of danger after four hours' artificial respiration.

**The Rochester Pathological Society,** April 8. Dr. A. A. Thibaudeau of Buffalo gave a paper on Practical Value of Serodiagnosis.

**Rochester Academy of Medicine,** Section 4, April 14. The Thymus Question, Dr. C. W. Hennington; Sarcoma of the Mediastium, Dr. J. W. McGill; Some Observations on Cases of Hyperthyroidism, Dr. J. M. Swan.

**Buffalo Academy of Medicine,** Section of Pathology, March 24, The Optic Nerve in Disease, Dr. W. L. Phillips; Visual Paths in Disease, Dr. F. Park Lewis.

Section of Surgery, April 7, Preliminary Report on the Results of Heliotherapy on Surgical Tuberculosis at the J. N. Adam Memorial Hospital, Drs. Hyde and LoGrasso of Perrysburg. (Illustrated by stereopticon and moving pictures.) Clinical Results in Operations on Tumors of the Bladder by Various Operators for the Past 15 Years. Review of 1,588 cases. Dr. James Gardner. This was an open meeting to which ladies were invited. A collation was served.

Medical Section, April 14, Vascular Phenomena, Dr. Henry C. Buswell.

Section of Obstetrics and Gynaecology, April 21, Status of Pubiotomy as an Obstetrical Procedure, Dr. J. Whitridge Williams, Baltimore.

---

The regular meeting of the **Medical Society of the County of Erie** was held Monday evening, April 19, at 8.45 at the University of Buffalo. In the absence of President Hurd, First Vice-President Dr. Franklin W. Barrows presided. The secretary read the minutes of the Council meeting of the Society held February 15, and also the minutes of the Council meeting held February 23, both of which were approved as read.

The secretary presented a letter from Dr. Wisner R. Townsend, secretary of the State Society, calling attention to Assembly bill 1107, Int. 1020 and Senate Int. 868, to amend the Judiciary law in relation to the appointment of examining physicians in criminal or special proceedings, had passed both houses at Albany and it was now before the Governor for his consideration. As this bill has been favored by the State Society it was approved on motion of Dr. Bleau and Dr. Coakley, and the secretary instructed to write to the Governor accordingly.

The secretary presented a letter from Ruby H. Etches, secretary of the Mothers Club of Trinity Episcopal Church, Buffalo, stating that the club has placed at the disposal of any physician a number of packages of sterile goods, including such as are most commonly used in obstetric cases, and also that such packages may be had by application at Police Headquarters.

On motion of Dr. McKee the secretary was requested to acknowledge the receipt of this letter and to express the appreciation of the County Society of the action of this Club.

At the suggestion of Health Commissioner Fronczak the secretary was requested to ask the Mothers Club of Trinity Episcopal church to kindly co-operate to whatever extent possible in this respect with the Social Welfare Center conducted by the Department of Health and others at 1071 Grant Street.

The secretary presented a communication from the New York Academy of Medicine requesting the Society to take favorable action on the request to transfer the quarantine station at the port of New York from the control of New York State to that of the Public Health Service.

On motion of Dr. Coakley, seconded by Dr. Fronczak, a resolution was adopted approving said transfer.

Dr. Bennet said that in view of the fact that the New York State Society will hold its next meeting in Buffalo, and as one of our fellow-members is the esteemed president of our State Society, it is essential that he have the undivided support of the delegation from this county. He therefore moved the following resolution, which was carrried:

"In order that the Erie County Medical Society shall exert its full influence in the council of the State Society and to avoid the possibility of a divided delegation neutralizing that influence, be it therefore Resolved: That the delegates of the Medical Society of the County of Erie be instructed to vote as a unit on all questions as determined by caucus." This resolution was uanimously adopted.

Dr. Fronczak brought up the subject of bills now before the State legislature relative to the New York State Department of Health. He moved that the Society disapprove of Assembly bill No. 1943, which requires that all regulations and sanitary code of the Public Health Council must be approved by the legislature before becoming effective. Carried.

Dr. Fronczak then moved to disapprove of Assembly bill No. 1942, which prescribes qualifications through the legislature of health officers, public health nurses and sanitary supervisors. Carried.

Dr. Fronczak then moved to disapprove of Assembly bill No. 1561, which requires the State Commissioner of Health and directors of divisions to devote their entire time to their duties.

Dr. Bennet said that if the salaries for these offices were inadequate they ought to be made sufficient. He therefore moved an amendment that the Society approve Bill No. 1561. The amendment was seconded by Dr. McKee. Dr. Jones stated that he would favor the amendment on condition that the Society indicates its disapproval of the small amount of salary paid the State Health Commissioner, and that his salary be made adequate. Drs. Lytle, Woodruff and VanPeyma spoke along the same lines. The amendment was then carried.

On motion of Dr. Bennet the secretary was directed to notify the chairman of the Public Health Committee of the legislature of the action of the Society.

Dr. McKee moved that the president appoint a committee of two to draft a suitable resolution expressing the sympathy of this Society on the sad affliction which has befallen one of our oldest members, Dr. Henry R. Hopkins. The president appointed as such committee Dr. Van Peyma and Dr. A. A. Jones, who reported the following: "At the regular meeting of the Medical Society of the County of Erie held April 19, 1915, the following memorial was unanimously adopted:

"This Society has learned of the great affliction which has befallen one of our oldest and most distinguished members, Dr. Henry R. Hopkins, in the death of his devoted and beloved wife, and we hereby express our deep sympathy to Dr. Hopkins and his daughters in their sad affliction."

The report of the committee was unanimously adopted, and on motion the secretary was directed to send a copy of same to Dr. Hopkins and his family.

The scientific part of the program consisted of a discussion of the diagnosis of Cardiac Irregularities by Dr. Clayton W. Greene. The treatment of Cardiac Irregularities was to have been discussed by Dr. Nelson G. Russell, but Dr. Greene announced that Dr. Russell had been unavoidably detained out of town and had telegraphed his regrets. Dr. Greene therefore covered the entire subject which had been assigned to both in such an admirable manner as to elicit the heartiest commendation of a number of members present.

The Society then adjourned.

FRANKLIN C. GRAM, Secretary.

